# Telomere-to-telomere genome assembly of pumpkin (*Cucurbita moschata*) facilitates QTL identification of fruit traits

**DOI:** 10.1093/hr/uhag141

**Published:** 2026-04-16

**Authors:** Yiming Lu, Jiaying Liu, Liting Deng, Xiaoshan Wei, Jianbin Zhang, Haibin Wu, Jianning Luo, Xiaoxi Liu, Gangjun Zhao, Hao Gong, Xiaoming Zheng, Qiusheng Kong, Junxing Li

**Affiliations:** Guangdong Key Laboratory for New Technology Research of Vegetables, Vegetable Research Institute, Guangdong Academy of Agricultural Sciences, Guangzhou 510640, China; National Key Laboratory for Germplasm Innovation & Utilization of Horticultural Crops, College of Horticulture & Forestry Sciences, Huazhong Agricultural University, Wuhan 430070, China; Guangdong Key Laboratory for New Technology Research of Vegetables, Vegetable Research Institute, Guangdong Academy of Agricultural Sciences, Guangzhou 510640, China; National Key Laboratory for Germplasm Innovation & Utilization of Horticultural Crops, College of Horticulture & Forestry Sciences, Huazhong Agricultural University, Wuhan 430070, China; Guangdong Key Laboratory for New Technology Research of Vegetables, Vegetable Research Institute, Guangdong Academy of Agricultural Sciences, Guangzhou 510640, China; Guangdong Key Laboratory for New Technology Research of Vegetables, Vegetable Research Institute, Guangdong Academy of Agricultural Sciences, Guangzhou 510640, China; Guangdong Key Laboratory for New Technology Research of Vegetables, Vegetable Research Institute, Guangdong Academy of Agricultural Sciences, Guangzhou 510640, China; Guangdong Key Laboratory for New Technology Research of Vegetables, Vegetable Research Institute, Guangdong Academy of Agricultural Sciences, Guangzhou 510640, China; Guangdong Key Laboratory for New Technology Research of Vegetables, Vegetable Research Institute, Guangdong Academy of Agricultural Sciences, Guangzhou 510640, China; Guangdong Key Laboratory for New Technology Research of Vegetables, Vegetable Research Institute, Guangdong Academy of Agricultural Sciences, Guangzhou 510640, China; Guangdong Key Laboratory for New Technology Research of Vegetables, Vegetable Research Institute, Guangdong Academy of Agricultural Sciences, Guangzhou 510640, China; Guangdong Key Laboratory for New Technology Research of Vegetables, Vegetable Research Institute, Guangdong Academy of Agricultural Sciences, Guangzhou 510640, China; Guangdong Key Laboratory for New Technology Research of Vegetables, Vegetable Research Institute, Guangdong Academy of Agricultural Sciences, Guangzhou 510640, China; National Key Laboratory for Germplasm Innovation & Utilization of Horticultural Crops, College of Horticulture & Forestry Sciences, Huazhong Agricultural University, Wuhan 430070, China; Guangdong Key Laboratory for New Technology Research of Vegetables, Vegetable Research Institute, Guangdong Academy of Agricultural Sciences, Guangzhou 510640, China

## Abstract

*Cucurbita moschata*, commonly known as pumpkin, is highly valued for its nutritional content, stress tolerance, fruit phenotypic diversity, excellent characteristics of easy-planting and transportation, and long shelf-life, which makes it recommended as an essential crop for the mitigation of food and nutritional challenges. However, the absence of a high-quality reference genome hinders genetic improvement studies on pumpkins. Here, we successfully assembled the telomere-to-telomere (T2T) genome of *C. moschata* L. (2*n* = 40) with a total size of 289.6 Mb (Contig N50 = 11.2 Mb). It comprises all 40 telomeres, 8 gaps, and 29 901 protein-coding genes (BUSCO = 97.9%), and half of the chromosomes are composed of a single contig. Additionally, to investigate the genetic basis of fruit traits in pumpkin, we developed an F_2_ genetic population consisting of 200 individuals and constructed a high-density genetic linkage map using the assembled genome as the reference. Quantitative trait locus (QTL) mapping of 20 fruit traits was applied. An AUX/IAA gene and a β-tubulin gene were identified as candidate genes regulating longitudinal flesh thickness and transverse/longitudinal diameter ratio, respectively. In summary, the genome assembly for *C. moschata* and QTL mapping results provide valuable resources for molecular breeding and functional genomics studies in the pumpkin.

## Introduction

Pumpkin (*Cucurbita moschata*, 2*n* = 2*x* = 40), believed to originate from Central America [1], is one of the key ancestral *Cucurbita* species [2]. *C. moschata* is one of the earliest domesticated types [3] and has spread globally with the development of agriculture. The global production of *Cucurbita* spp., including pumpkin, squash, and gourds, exceeded 23.6 million tons with a harvested area of over 1.5 million hectares in 2023 (https://www.fao.org/). As a well-domesticated crop, it is characterized by high yield and stress tolerance [4]. These characteristics endow pumpkin with better adaptability compared to other major *Cucurbit* crops [5] and make it a great preference as rootstocks for grafting [6]. Pumpkin is also highly valued for its excellent edibility. Its fruit, tendrils, flowers, seeds, and young stems are all edible [7]. Additionally, it is nutrient-rich and beneficial for health. The fruit flesh is abundant in sugars, fatty acids, carotenoids (CA), phenolics, flavonoids, vitamins, polysaccharides, and amino acids [8], and the seeds are rich in protein, oil, and vitamins [9]. These attributes make pumpkin an important vegetable crop in markets and an essential raw material in the food industries [10]. Nowadays, pumpkin is globally cultivated due to the excellent characteristics of easy-planting and transportation, and long shelf-life, and has been recommended as an essential crop for the mitigation of food and nutritional challenges, especially in developing countries [7, 11].

Notably, pumpkin exhibits a high degree of phenotypic diversity, especially in the fruit-associated traits, including fruit shape, size, weight, and colour [12, 13]. In recent years, research on the biological mechanisms underlying the pumpkin fruit development has gained increasing attention from both the scientific community and related industries. These studies provide valuable insights for breeding new cultivars with improved properties. However, due to the large size of pumpkin plants, the breeding process is often time-consuming and resource-intensive [7]. It is urgently necessary to enhance the breeding efficiency through advanced genomics.

Telomere-to-telomere (T2T) or complete genome assemblies facilitate the analysis of genome structure and provide essential resources for genetic improvement and molecular breeding [14, 15]. Within the *Cucurbita* genus, the genomes of *C. pepo* (MU-CU-16) [16]*, C. argyrosperma* ssp. *argyrosperma* (SMH-JMG-627) [17]*, C. argyrosperma* ssp. *sororia* (Sliver-seed gourd) [18]*,* and *C. maxima* (HZAU) [19] have been consecutively published. The genome of *C. moschata* (Rifu) was first *de novo* assembled and released in 2017 [20]. However, the use of short-read sequencing technology resulted in numerous gaps, thereby limiting the completeness and continuity of the genome. The fragmented assemblies may lead to the omission or inaccurate annotation of important genes and functional regions. With the advancement of third-generation sequencing technologies, chromosome conformation capture techniques, and assembly algorithms, the quality of genome assemblies has been significantly improved, making the T2T genome assembly feasible [21]. Given the economic and biological importance of *C. moschata*, it is urgent to assemble a high-quality reference genome with improved accuracy, continuity, and completeness to support the pumpkin research.

Gene and genome duplications are fundamental mechanisms driving genome evolution [22], with duplication events including whole-genome duplication (WGD), tandem duplication (TD) and segmental duplication (SD) [23]. In plant genomes, SDs and other forms of gene duplication serve as forces for structural and functional evolution [24]. Duplicated genes often exhibit functional divergence and are preferentially retained when involved in stress responses and environmental adaptation [25]. In pumpkin, ancient WGD events followed by random gene loss (unbiased fractionation) have significantly shaped genome architecture, contributing to phenotypic variation and adaptive evolution [20]. Long-read sequencing technologies and high-quality genomes enable more accurate function characterizations of the specific genes and genome duplications, along with their roles in complex traits.

A complete genome also provides a solid foundation for functional gene studies and gene-phenotype association analyses. Identifying candidate genes that regulate key traits through quantitative trait loci (QTL) mapping is vital for the improvement of agronomic traits. Several studies have reported the QTL mapping in *C. moschata* [7, 26, 27]. However, these studies have mainly focused on fruit appearance traits, with limited attention given to fruit quality. In addition, comprehensive research on candidate genes associated with various pumpkin fruit traits remains scarce. The QTL mapping intervals based on earlier reference genomes may suffer from inaccuracies or incomplete functional annotations of candidate genes. Hence, the study selected the pumpkin inbred line YGX2019 (*C. moschata*) with excellent agronomic traits for sequencing and genome assembly, with the aims to construct a high-quality and near-complete reference genome, characterize its genomic features that include repetitive sequences and duplicated genes, and identify QTLs controlling key fruit traits and screen for candidate genes using a high-density genetic map. These efforts will provide a more reliable foundation for the genetic improvement and molecular breeding of pumpkins.

## Results

### The assembly of the YGX2019 genome

**Figure 1:**
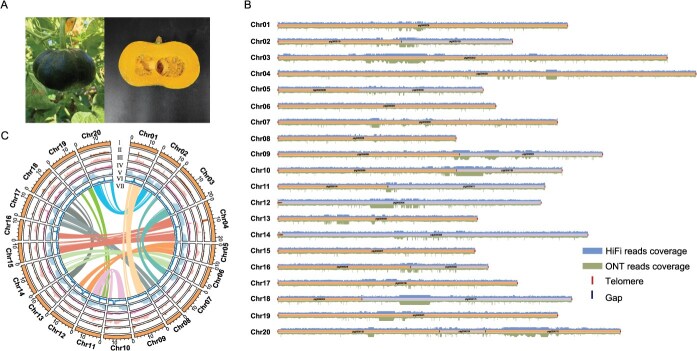
The YGX2019 genome assembly and annotation results. (**A**) The external and longitudinal sectional view of the YGX2019 fruit. (**B**) Overview of genome assembly statistics, displaying the HiFi and ONT read coverages, along with the positions of telomeres and gaps across chromosomes. (**C**) Circos plot of the YGX2019 genome annotation at a 100 Kb interval: I: Chromosomes. II: Density of non-coding RNA genes. III: Density of LTR-Gypsy elements. IV: Density of LTR-Copia elements. V: Density of annotated genes. VI: GC content. VII: Genome self-synteny, showing only the chromosomes with the highest synteny.

The pumpkin inbred line YGX2019 with excellent agronomic traits was selected for sequencing and genome assembly (Fig. 1A). The Illumina sequencing generated 19.8 Gb paired-end reads (150 bp), which were used to assess the genome characteristics. Based on the k-mer analysis (*k* = 21), the genome was estimated to be approximately 279.5 Mb with a heterozygosity of 0.127%. Therefore, it was unnecessary to separate the genome into two haplotypes (Fig. S1). To achieve a high-quality genome assembly, a total of 77.3× sequencing depth of PacBio HiFi reads and 63.1× ultra-long ONT reads were generated (Table S1). The tools of Hifiasm, Nexdenovo, and HiCanu were employed for contig assembly. Hifiasm utilized both the HiFi and ONT data resulted in 80 contigs (301 Mb, N50 = 12.8 Mb, longest contig = 25.8 Mb). NextDenovo used the ONT data produced 74 contigs totaling 279 Mb (N50 = 10.2 Mb, longest contig = 16.3 Mb). HiCanu that utilized the HiFi data generated 584 contigs totaling 281 Mb (N50 = 4.5 Mb, longest contig = 11.6 Mb) (Fig. S2, Table S2).

Given the highest continuity, the contigs generated by Hifiasm were selected for further scaffolding. The Juicer and 3D-DNA pipelines were utilized to scaffold contigs and anchor them onto pseudo-chromosomes using the Hi-C data (135.6×), and then the orders and orientations of contigs were manually corrected in the Juicebox. During the preliminary scaffolding stage, 26 contigs were anchored onto 20 pseudo-chromosomes. Following, two contigs (ptg000005l and ptg000009l) were found to span two chromosomes each (ptg000005l corresponded to Chr05 and Chr12, and ptg000009l to Chr10 and Chr14) (Table S3). After manual adjustment, these contigs were divided into chromosome-specific segments, resulting in a final total of 28 contigs anchored onto 20 pseudo-chromosomes, with a total size of 288.7 Mb and an anchoring ratio of 95.8% (Fig. S3). Notably, one contig for one chromosome was achieved for 13 pseudo-chromosomes, resulting in gap-free assemblies (Table S3). The remaining seven chromosomes contained eight gaps. Further analysis found that three chromosomes lacked one telomere each. To fill these gaps and complete the missing telomeres, contigs generated by HiCanu and NextDenovo were aligned with the assembled chromosomes. The contigs with good alignment and spanning the gaps were used to close the gaps (Fig. S4). Similarly, the missing telomeres were added using contigs with good alignment and telomeric sequences from contigs, finally completing all missing telomeres without filling the gaps (Fig. S5, Tables S4 and S5). Ultimately, a near-complete genome with a size of 289.6 Mb was assembled for YGX2019 (Fig. 1B). The BUSCO value reached 97.9% and the QV value was 50.2, indicating the excellent integrity and accuracy of the assembly (Table 1).

**Table 1:** Comparison of genomic features of the YGX2019 and Rifu genome assemblies.

**Genome features**	**YGX2019**	**Rifu**
Genome size (bp)	289 654 587	238 601 125
Sequencing technology	HiFi + ONT + Hi-C + Illumina	Illumina
Number of contigs	28	10 140
Contig N50 (bp)	11 203 200	43 566
Contig L50	10	1607
Average contig length (bp)	10 343 862	23 034
Largest contig (bp)	22 321 440	292 205
Smallest contig (bp)	2 444 691	68
Number of telomeres	40	4
Number of gaps	8	10 120
QV	50.2	
Base accuracy rate (%)	99.9763	
BUSCO (%)	97.9	97.8
GC (%)	36.65	36.46

### Genome annotation

A total of 142.6 Mb (49.24%) of the genome was identified as repetitive sequences, with long terminal repeat (LTR) retrotransposons being the most abundant, accounting for 75.5 Mb (26.06%). Among these, Copia elements constituted 10.70%, and Gypsy elements 9.76% (Table S6). The non-coding RNA genes, including tRNAs, miRNAs, and rRNAs, were also identified (Fig. 1C, Table S7). After softmask of the repetitive sequences, annotations on gene structures were performed using BRAKER3 based on *ab initio* prediction, homologous proteins and RNA-seq data. After filtering out genes lacking start or stop codons, a total of 29 901 protein-coding genes were annotated, with an average length of 2982 bp, among which 4054 genes exhibited alternative splicing. By searching the database, including the NR, Uniprot_sprot, Uniprot_trembl, eggNOG, and Interproscan, a total of 29 251 genes (97.8%) were functionally annotated (Table S8). The extracted protein sequences based on the annotated genes exhibited 97.9% BUSCO completeness.

### Genome quality assessment

**Figure 2:**
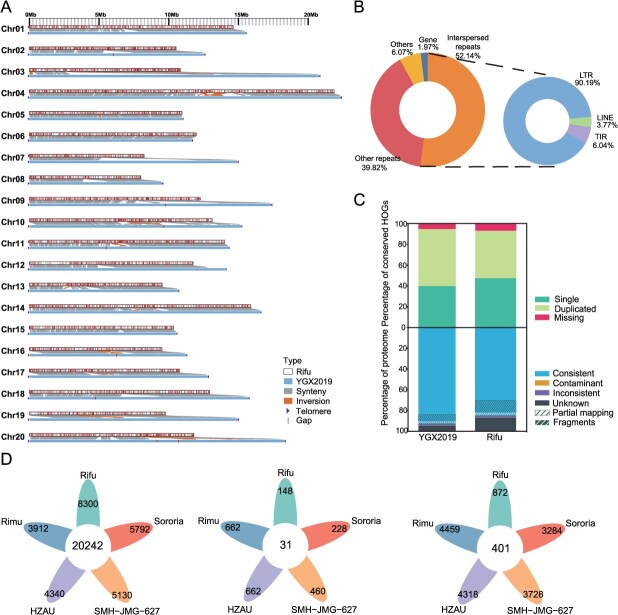
Genome quality assessment. (**A**) Synteny analysis between YGX2019 and the published *C. moschata* (Rifu) genome. (**B**) Composition of YGX2019 genome-specific sequences. The left doughnut chart shows the proportions of various sequence types within the genome, while the right doughnut chart provides a further classification of interspersed repeats, detailing their subcategories. (**C**) Completeness of proteome annotations for YGX2019 and Rifu. The top part presents the results of the completeness assessment. The bottom part shows the results of the consistency assessment. (**D**) Flower plots of the valid (left), unmapped (middle) and invalid (right) mapped genes between the YGX2019 genome and the other five published pumpkin genomes.

YGX2019 displays significant improvement in genome assembly continuity and completeness compared to *C. moschata* reference genome (Rifu). The contig N50 increased from 43.5 Kb to 11.2 Mb. The number of identified telomeres increased from 4 to 40, and the quantity of gaps dramatically decreased from 10 120 to 8, indicating that YGX2019 genome exhibits higher completeness (Fig. 2A). The QV value of 50.2 and base accuracy of 99.9763% were calculated using Illumina sequencing data, demonstrating the exceptional accuracy of the YGX2019 genome (Table 1).

Interestingly, YGX2019 has a longer average CDS length (236.1 vs 216.9 bp), less average exons per gene (5.6 vs 5.8), and shorter average exon length (236 vs 255 bp). These differences may reflect evolutionary divergence, gene structure variation, or discrepancies in annotation methods (Table S9). Regarding to the gene annotation, there were 32 023 genes annotated in the Rifu genome and a total of 27 513 genes were functionally annotated, resulting in a functional annotation rate of 85.9%. The relatively low annotation rate may be attributed to the incomplete genome and a substantial number of pseudogenes. The collinearity analysis revealed collinear regions and structural variations (SVs) between two genomes, with collinear region lengths of 289.6 Mb (86.1%) in the YGX2019 genome and 238.6 Mb (98.1%) in the Rifu genome. A total of 13 inversions were identified between two genomes, covering 1.7 and 1.8 Mb, respectively (Fig. 2A).

Subsequently, the composition of newly assembled regions was analyzed. Compared with Rifu, 91.96% (42.6 Mb) of the additional sequences in the YGX2019 genome consist of repetitive sequences. Interspersed repeats make up 52.14% (24.1 Mb), and LTRs account for 90.19% (21.7 Mb) (Fig. 2B). Only 1.97% (0.91 Mb) of the additional sequences were gene sequences and encompass 415 genes, including those involved in stress resistance (for instance *Mos_3HG001740* and *Mos_11HG007240*) and flowering time regulation (for instance *Mos_4HG018950*). The result indicates that the incompleteness of the Rifu genome was mainly due to unassembled repetitive sequences, especially transposable elements (TEs), which the YGX2019 genome assembly successfully addressed.

To further evaluate annotation quality, the completeness of the predicted proteome was assessed using OMArk. The YGX2019 genome demonstrated superior completeness (94.99% vs 93.12%) and consistency (92.28% vs 84.55%) compared to Rifu, and had fewer proteins of unknown function (5.70% vs 13.39%) (Fig. 2C). Compared to previously published pumpkin genomes, YGX2019 showed fewer missing proteins (Table S10). These findings underscore the high quality and comprehensiveness of gene annotations in YGX2019. Mapping the genes of the YGX2019 genome to five other pumpkin genomes revealed that 20 242 valid and shared mapping genes. The number of genes mapped to *C. maxima* (Rimu) was the lowest (24154), while the highest was to *C. moschata* (Rifu) (28542). There were 31 shared unmapped genes and 401 shared invalid mapping genes, which may represent unique genes in YGX2019. Among the five genomes, Rimu and HZAU exhibited the highest number of unmapped genes (693), accounting for 2.3% of the YGX2019 gene counts. In contrast, Rimu harboured the most invalid mapping genes (4860), constituting 16.2% of the YGX2019 genome (Fig. 2D). In conclusion, the YGX2019 genome demonstrates superior assembly quality, annotation completeness, and accuracy, providing a reliable genomic resource for genetic and breeding research in pumpkin.

### Genomic evolution and comparative genomic analysis of *Cucurbita*

**Figure 3:**
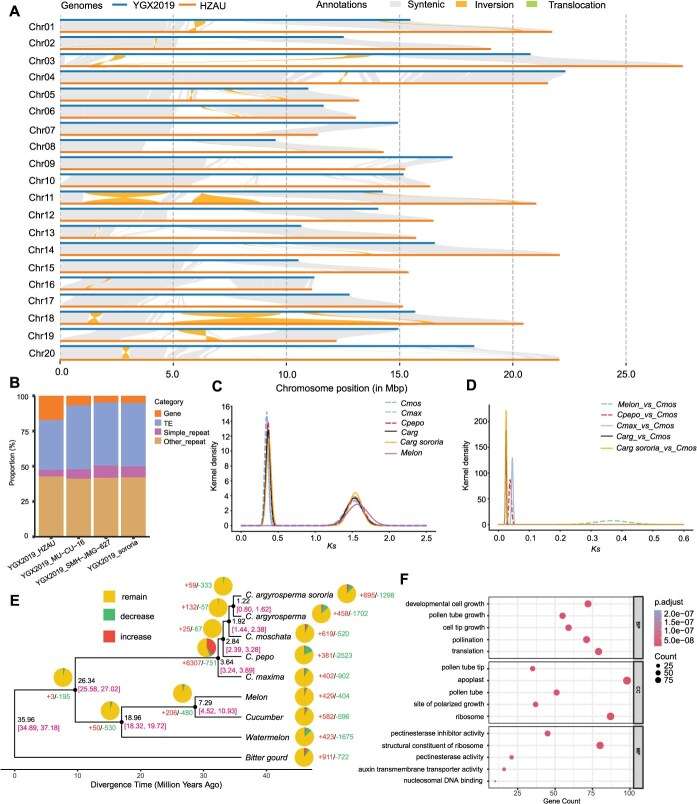
Genomic evolution and comparative genomic analysis of *Cucurbita* (**A**) Genomic synteny and structural variations between *C. moschata* (YGX2019) and *C. maxima* (HZAU). (**B**) Proportions of unaligned sequence types between YGX2019 and other published *Cucurbita* genomes. (**C)** Distribution of synonymous substitution rates (*K_s_*) of paralogous gene pairs within five *Cucurbita* species and melon, highlighting WGD events. *Cmos*: *Cucurbita moschata*; *Cmax*: *Cucurbita maxima*; *Cpepo*: *Cucurbita pepo*; *Carg*: *Cucurbita argyrosperma*; *Carg sororia*: *Cucurbita argyrosperma* subsp. *sororia* (**D)**  *K_s_* distribution of orthologous gene pairs between *C. moschata* and other species, indicating speciation divergence times. (**E)** Phylogenetic tree and divergence times of nine sequenced plant species. Pie charts on the nodes and branches show the proportions of gene families that expanded (+), contracted (-), or remained conserved. Numbers at the nodes indicate the estimated divergence time (Mya), with 95% highest posterior density (HPD) intervals shown in brackets. Numbers next to pie charts show the exact count of expanded and contracted gene families. (**F)** GO enrichment analysis of the significantly expanded gene families in the *Cucurbita* common ancestor.

To comprehensively elucidate the genetic variations driving the species-specific divergence of *C. moschata*, we performed comparative genomic analyses between YGX2019 assembly and four published *Cucurbita* genomes (*C. maxima* HZAU, *C. pepo* MU-CU-16, *C. argyrosperma* SMH-JMG-627, and *C. argyrosperma* subsp*. sororia* sororia). Whole-genome synteny alignments revealed complex SVs across the *Cucurbita* genus (Figs 3A and S6). Specifically, the comparison between YGX2019 and the complete *C. maxima* genome (HZAU) identified 34 inversions, 62 duplications, and 33 translocations (Table S11). Extending the analysis to other *Cucurbita* species demonstrated that genome structure shows highly dynamic within the genus. For instance, the alignment with *C. pepo* (MU-CU-16) uncovered 28 inversions, 50 duplications, and 19 translocations (Table S11). Similarly, comparisons with SMH-JMG-627 and the wild relative sororia also revealed large-scale SVs (Table S11). These extensive genomic rearrangements underscore the necessity of utilizing multiple reference genomes to capture the evolutionary dynamics of *Cucurbita*.

Furthermore, we identified a substantial proportion of unaligned genomic regions across all comparisons. In YGX2019, the unaligned sequences accounted for 29.17%, 30.34%, 22.60%, and 22.05% of the genome assembly when compared to HZAU, MU-CU-16, SMH-JMG-627, and sororia, respectively (Table S11). These sequences are primarily distributed in the central regions of chromosomes, suggesting substantial variation in centromeric sequences between the species (Fig. 3A, Fig. S6, Table S11). These structurally divergent regions encompass a number of protein-coding genes (ranging from 628 to 1566 YGX2019 genes) that may contribute to species-specific phenotypic traits and adaptation. To investigate the sequence composition and functional implications of the unaligned regions, we annotated the distinct genomic elements. Consistent across all comparisons, TEs and repetitive sequences constituted the majority of these unaligned regions, collectively accounting for about 80% of the total unaligned bases (Fig. 3B; Table S12). It indicates that the differential accumulation or deletion of repetitive elements is the primary driver of genomic SVs and size differences among *Cucurbita* species. Only a minor fraction of these unaligned regions contained protein-coding genes. Nevertheless, these genes may harbor signatures of species-specific adaptations. GO enrichment analysis revealed that the unaligned genes between YGX2019 and other distinct species (*C. maxima* HZAU and *C. pepo* MU-CU-16) were prominently enriched in secondary metabolite biosynthesis and defense responses to herbivores (Fig. S7A and B). In contrast, genes located in the unaligned regions against the SMH-JMG-627 and the wild relative sororia were mainly associated with environmental stress responses and enzymatic regulation (Fig. S7C and D). Together, these findings suggest that the dynamic unaligned genomic regions are not merely non-functional variations, but could potentially contribute to defense mechanisms during the evolution of *C. moschata*.

To trace the evolutionary history and polyploidization events of *C. moschata*, we calculated the synonymous substitution rate (*K_s_*) distributions for paralogous and orthologous gene pairs among the published genomes. The *K_s_* distribution of paralogs within the five *Cucurbita* genomes revealed two distinct peaks (Fig. 3C). The older peak, located at *K_s_* ≈ 1.5, was shared among all analyzed species including melon, representing the ancient core eudicot *γ* hexaploidization event. Notably, a secondary, prominent peak at *K_s_* ≈ 0.35 was exclusively identified in the five *Cucurbita* genomes but was absent in melon. This suggests a recent *Cucurbita*-specific WGD event occurred in the common ancestor of the genus, which is highly consistent with previous reports [20]. To further determine the timing of these evolutionary events, we analyzed the *K_s_* distributions of orthologous gene pairs between YGX2019 and other species. The orthologous *K_s_* peak between *C. moschata* and melon largely overlapped with the recent WGD peak (*K_s_* ≈ 0.35) (Fig. 3D), indicating that the *Cucurbita*-specific WGD event occurred around the divergence of the Cucurbiteae (*Cucurbita*) and Benincaseae (melon, cucumber, watermelon) tribes. Conversely, the orthologous *K_s_* peaks among the *Cucurbita* species were extremely low (*K_s_* < 0.05), reflecting a very recent and rapid species radiation. These findings are supported by calibrated phylogenetic timetree (Fig. 3E). The divergence between the *Cucurbita* lineage and the Benincaseae tribe was estimated at approximately 26.34 million years ago (Mya). Subsequently, following the genus-specific WGD event, the *Cucurbita* common ancestor began to rapidly diversify around 3.64 Mya, with *C. moschata* separating from its sister lineages approximately 1.92 Mya.

Gene family expansion and contraction are crucial mechanisms driving phenotypic diversification. Based on our time-calibrated phylogeny (Fig. 3E), we investigated the gene family dynamics across the *Cucurbita* lineages. Notably, a massive gene family expansion was observed at the common ancestor node of the *Cucurbita* genus, with 6307 expanded and 751 contracted families. This burst of gene expansion is likely a direct genomic consequence of the recent WGD event. GO enrichment analysis on these ancestrally expanded genes showed significant enrichment in fundamental processes required for post-polyploidization cellular growth, such as translation and ribosome constitution (Fig. 3F). Furthermore, reproductive-related terms, for instance, pollination and pollen tube growth, were enriched, suggesting a potential role of these expanded genes in establishing reproductive isolation during early speciation. Following its divergence from sister lineages, *C. moschata* (YGX2019) experienced further lineage-specific gene family dynamics, including the expansion of 619 families and the contraction of 520 families (Fig. 3E). To explore how these specific genomic changes shaped the unique traits of *C. moschata*, we analyzed the GO enrichment of its expanded genes (Fig. S8). The YGX2019 specific expanded genes were distinctly enriched in defense-related pathways, for instance, the response to herbivores. The genomic signatures suggest that the lineage-specific expansion of gene families in *C. moschata* could potentially contribute to the environmental adaptability during its independent evolutionary trajectory.

### Genome and gene duplications in YGX2019

**Figure 4:**
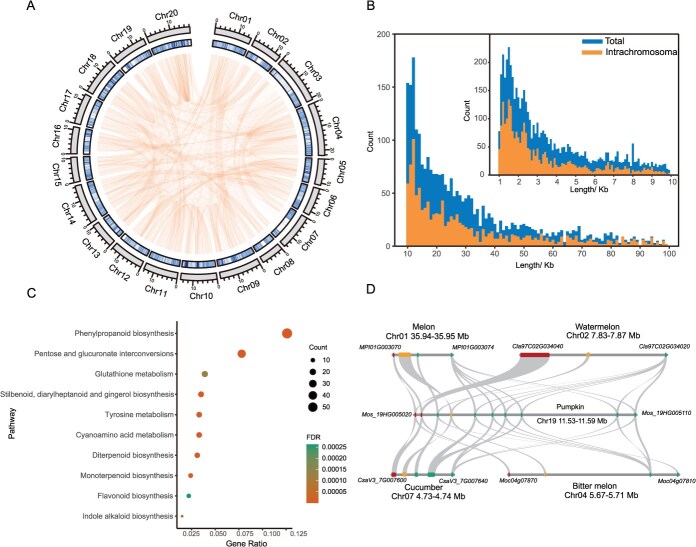
Genome and gene duplications analysis of the YGX2019 genome. (**A)** Heatmap in the circos plot indicates the density of the SD regions. The linked regions indicate the SD regions between chromosomes. (**B)** The distribution of SDs of different lengths. (**C)** The top 10 significant Kyoto Encyclopedia of Genes and Genomes (KEGG) pathways enriched in TD genes. The *P* values were corrected using the FDR method. (**D)** Copy number variation of a resistance gene cluster across diverse Cucurbitaceae species. The same gene orthogroup is indicated by the same color.

The near-complete genome provides the opportunity to examine the highly repetitive and complex regions, facilitating a deeper understanding of their structure and biological function. We first identified whole-genome SDs. A total of 8340 pairs of SDs were identified in the YGX2019 genome, with an aggregate length of 91.5 Mb. These SDs account for 31.6% of the total genome length (Fig. 4A), which surpassed the pear (10.7%) [24], asparagus bean (15.3%) [28], and rice (24.2%) [29]. The proportion is also higher than that of Rifu (68.6 Mb, 28.7%), indicating that the assembly using long reads contributes to more thorough identification of complex genomic structures.

The distribution of SDs showed substantial interchromosomal variation, with no significant correlation observed between SD abundance and chromosome length (Table S13). For instance, Chr20 contains the longest SDs (7.9 Mb) and the highest proportion (43.6%). In contrast, Chr08, despite having the shortest SDs (1.65 Mb), has a higher proportion (17.4%) than Chr02 (14.5%). Furthermore, SDs were found to be unevenly distributed within chromosomes. Low SD density was identified on Chr02, Chr07, Chr08, Chr12, Chr19, and high-density areas were discovered on Chr05 and Chr20. Regardless of SD length, the proportion of intrachromosomal SDs (48.7%, 4065/8340) is nearly equal to that of interchromosomal SDs (51.3%, 4275/8340), indicating the random distribution of SDs within the genome (Fig. 4B). The *C. moschata* genome underwent a WGD event, and the subsequent random loss of genes may explain the approximately equal number of SDs within and between chromosomes in the YGX2019 genome [20]. Among SDs ranging from 1 to 100 Kb in length, 49.8% are distributed within 1–3 Kb and 10–20 Kb ranges. The average length of intrachromosomal SDs (43 776 bp) significantly exceeds that of interchromosomal SDs (15 139 bp).

To elucidate the biological significance of duplicated genes in the pumpkin, we assigned a unique duplication type to each gene. We classified gene pairs as derived from SD, TD, dispersed duplications (DD), or proximal duplications (PD), to understand the different functions of various duplication modes. Among the total 29 901 genes, 25 580 were identified as duplicated genes, including 18 085 SD, 1861 TD, 5114 DD, and 519 PD. In comparison, 21 618 out of 32 023 genes in the Rifu genome were duplicated genes, comprising 15 355 SD, 1448 TD, 4139 DD, and 676 PD (Fig. S9). Kyoto Encyclopedia of Genes and Genomes (KEGG) enrichment analysis was performed on TD, DD, and PD genes. DD genes were enriched in primary metabolic pathways, including starch, sucrose (SU), and glycerolipid metabolism (Fig. S10A). TD and PD genes showed significant enrichment in the phenylpropanoid biosynthesis pathway, which is associated with disease resistance [30], having the highest gene count and significance (Figs 4C and S10B). Additionally, we identified a resistance gene cluster on Chr19 composed of tandemly duplicated genes. Analysis of homologous genes across the Cucurbitaceae family revealed variations in copy number, with pumpkin exhibiting the highest variation number. Except for *Moc04g07850* in bitter melon, these homologs were uniformly organized as tandem duplicates, indicating a common genomic feature in the family (Fig. 4D).

### Phenotypic analysis of F_2_ population

**Figure 5:**
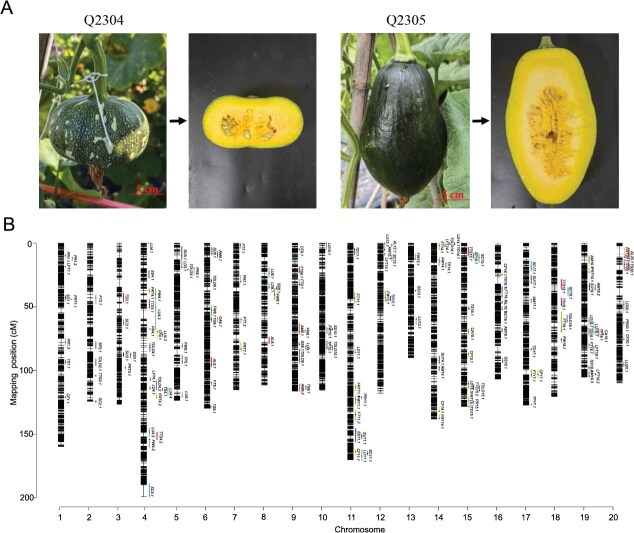
Locations of 165 QTLs in the YGX2019 genome. (**A)** The parental lines. (**B)** Genetic map. Black bands represent the bin markers on chromosomes. The circle in each interval represent the peak position.

To investigate the genetic basis of fruit traits, two distinct *C. moschata* inbred lines Q2304 (oblate/lantern shape) and Q2305 (long/olive shape) (Fig. 5A) were selected as parents to develop an F_2_ population, which provides a foundation for subsequent genetic map construction and QTL mapping. A total of 20 fruit traits were assessed, including fruit weight (FW), flesh thickness (FT), longitudinal flesh thickness (LFT), transverse diameter (TD), longitudinal diameter (LD), transverse/longitudinal diameter (TDLD) ratio, soluble solids (SS), total sugar (TS), fructose (FRT), SU, total starch (TTS), amylose (AM), amylopectin (AL), total pectin (TP), protopectin (PR), soluble pectin (SP), crude fibre (CF), lutein (LU), CA, and skin colour (SC) (Table S14). Except for TD, SS, CF, and LU, the coefficients of variation for other traits exceeded 20%, indicating the phenotypic diversity of the genetic population. Apart from SC, all other traits exhibited the characteristics of quantitative traits (Fig. S11), suggesting that these traits are suitable for QTL mapping studies. Correlation analysis revealed that TDLD was significantly and negatively correlated with both LD and LFT. The positive correlation between TD and FW was stronger than that between LD and FW, suggesting that TD had a more significant contribution to FW than LD. Additionally, TS showed a significant positive correlation with TTS, indicating that TS content is primarily determined by starch content (Fig. S11).

### Whole genome sequencing and construction of genetic maps

To determine the genotype of two parent lines and 200 F_2_ individuals, a total of 202 WGS libraries were prepared. The clean reads for the parental lines of Q2304 and Q2305 were 9.2 and 9.9 Gb, representing 29.7× and 28.2× sequencing depth, respectively. The clean reads generated by the F_2_ individuals were 11.12 Gb on average, ranging from 2.38 to 12.40 Gb. The coverage of the F_2_ individuals was 94.46%, varying from 58.28% to 98.20% (Table S15). The clean reads were aligned to the YGX2019 genome using bwa-mem2, achieving successful mapping rates ranging from 99.19% to 99.78%. Variant calling with BCFtools identified a total of 2 401 698 variants, including 2 044 832 SNPs and 356 866 InDels. SNPs were filtered based on parental genotypes using GATK SelectVariants, and 621 806 SNPs were obtained for downstream analysis. The SNPs within non-recombinant regions were grouped into bins for further analysis.

The high-density genetic map consists of a total of 7213 bin markers, covering a genetic distance of 2538.269 cM (Fig. 5B, Table S16). The average marker distance is 0.353 cM, with a maximum spacing of 9.358 cM. The number of markers per chromosome ranges from 284 (Chr16) to 533 (Chr11), with genetic lengths varying from 90.005 cM (Chr13) to 200.429 cM (Chr04). The average marker spacing ranges from 0.309 cM (Chr13) to 0.423 cM (Chr04), and the maximum distance between adjacent markers varies from 1.751 cM (Chr09, Chr12) to 9.358 cM (Chr04). Notably, the maximum distance between adjacent markers on chromosomes other than Chr04, Chr16, and Chr20 is less than 5 cM. There was a strong concordance between the genetic marker positions and the physical positions, validating the accuracy of the genetic map (Fig. S12). These characteristics demonstrate the high quality and reliability of the genetic map, providing a solid foundation for subsequent gene mapping studies.

### Identification of QTLs and candidate genes

In the study, a total of 165 QTLs associated with 20 key agronomic traits were identified using the composite interval mapping (CIM) method (Table S17). These traits could be categorized into four groups which were separately associated with fruit morphology (FW, FT, LFT, TD, LD, and TDLD), sugar and starch content (SS, TS, FRT, SU, TTS, AM, and AL), pectin and fibre (TP, PR, SP, and CF), and pigments (LU, CA, and SC). FW and FT had the maximum number of QTLs (11 QTLs each) found, explaining 61.60% and 47.62% phenotypic variance explained (PVE), respectively. All QTLs for FW exhibited additive and dominant effects with absolute values exceeding 10, suggesting that FW may be strongly regulated by certain genes. Nine QTLs for LD and 10 for TDLD were identified, which explained 65.70% and 60.60% of the total PVE, respectively. LFT and TD had the fewest QTLs (seven QTLs each) identified, explaining 76.16% and 49.73% PVE, respectively.

**Figure 6:**
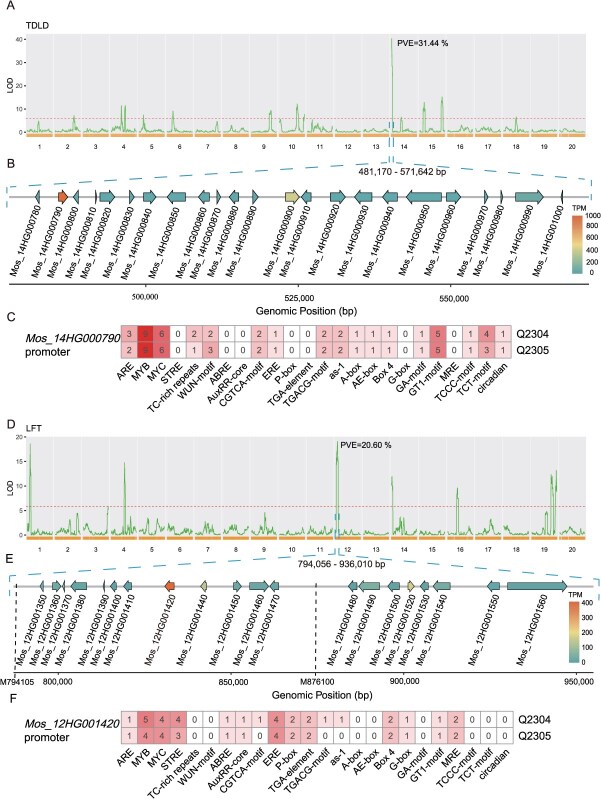
QTL mapping of TDLD and LFT. (**A)** QTLs for TDLD. The red line represents the LOD threshold. (**B)** Genes within the *TDLD14.1* interval. Different colors represent transcript abundance in young fruits of YGX2019 (TPM value). (**C)** Variations of cis-regulatory elements in the upstream 2 kb of the transcription start site between the parental lines. (**D)** QTLs for LFT, with the red line representing the LOD threshold. (**E)** Genes within the *LFT12.1* interval. Different colors represent transcript abundance in fruit (TPM value). The candidate region was narrowed to an interval of 82 kb which was bounded by the KASP markers M794105 and M876100 (indicated by vertical black dashed lines). (**F)** Cis-regulatory elements variations within the 2 kb region upstream of the transcription start site between the parental lines.

A major-effect QTL *TDLD14.1*, which explained 31.44% of the PVE, was identified (Fig. 6A, Table S19). To validate the QTL, we developed a co-dominant InDel marker based on resequencing data from the parental lines. The marker targets a 28-bp insertion that presents in the flat-shaped parent (Q2304) but absents in the oval-shaped parent (Q2305). The InDel sequences of Q2304 and Q2305 were AGTTGCATTTCCATTAGATGATAAAAGTT and A, respectively. Primers were designed to detect the InDel and used for amplification analysis of two parental lines and F_2_ population (Table S18). As expected, amplification yielded a 233-bp fragment for Q2304, a 205-bp fragment for Q2305, and both fragments for the F_1_ generation (Fig. S13). Among the 113 F_2_ individuals, 93 individuals had genotypes that were consistent with phenotypes (Fig. S13), resulting in a genotype-based prediction accuracy of the phenotype of 82.3%. The high accuracy not only validates the *TDLD14.1* locus but also provides a valuable tool for marker-assisted selection (MAS) in pumpkin breeding.

Additionally, multiple QTL overlaps were observed. For instance, *TDLD14.1* overlapped with *FT14.1, LFT14.1*, and *TD14.1*. And, *LD4.1* and *LD15.1* overlapped with *TDLD4.1* and *TDLD15.1*, respectively. The results suggest that these traits could be regulated by shared genes. Within *TDLD14.1*, we identified 23 genes (Fig. 6B, Table S19). Seven of them (*Mos_14HG000780*-*Mos_14HG000840*) were located within the overlapped interval. The *Mos_14HG000790* is predicted to encode a β-tubulin, a protein essential for controlling the direction of cell elongation and cellulose deposition via cortical microtubules [31]. To identify the most likely candidate within the target region, we analyzed the developmental transcriptome of Q2304 fruits from 2 days before flowering to 5 days after flowering. The results revealed that *Mos_14HG000790* was the most dominantly expressed gene across the *TDLD14.1* during these critical early stages of fruit shape determination (Fig. S14A). Crucially, to verify the association between gene expression and fruit shape, we analyzed its expression in multiple pumpkin varieties with contrasting phenotypes and found that *Mos_14HG000790* expression was significantly higher in varieties with oblate/lantern shapes (Q2302–Q2304) compared to those with long/olive shapes (Q2305–Q2307) (Fig. S15A). In addition, *Mos_14HG000790* exhibited its highest expression in fruits and young leaves (Fig. S15B). Furthermore, nine InDels and 59 SNPs identified in its promoter region (Fig. S16A). These variations lead to differences in cis-regulatory elements between two parental lines (Fig. 6C). Collectively, these results suggest that higher expression of this β-tubulin gene, likely driven by promoter variations, promotes the formation of oblate fruit shapes.

The *LFT12.1* is another major-effect QTL. To validate the locus and pinpoint the causal gene, we performed fine mapping using KASP markers derived from parental SNPs. By analyzing recombination events in five F_2_ individuals (S011, S089, S109, S138, and S199), we successfully narrowed the candidate region to a precise 82 kb interval bounded by markers M794105 and M876100 (Figs S17 and S18, Table S20). This fine-mapping reduced the number of candidate genes in this region from 20 to 12. Within the refined interval containing 12 genes, developmental transcriptome profiling of Q2304 fruits showed that most genes were barely expressed, while *Mos_12HG001420* exhibited exceptionally high and active transcription (Fig. S14B). The gene *Mos_12HG001420*, which encodes the auxin-responsive protein IAA9, is located precisely within the refined interval (Fig. 6D–E, Table S17, Table S19). Moreover, in a panel of diverse pumpkin varieties, the expression of *Mos_12HG001420* was found to be higher in varieties with oblate/lantern shapes (Fig. S15C). Additionally, *Mos_12HG001420* was highly expressed in young fruits (Fig. S15D). It has been reported that downregulation of the homologous IAA17 in tomato results in fruits with larger diameters by repressing cell elongation [32]. In addition, variations were also found in the promoter region (four InDels and 27 SNPs, Fig. S16B), which may modulate *Mos_12HG001420* expression levels, thereby determining FT.

Regarding the sugar and starch content traits, ten, nine, ten, seven, seven, ten, and five QTLs were separately identified for SS (PVE, 56.68%), TS (PVE, 65.26%), FRT (PVE, 75.05%), SU (PVE, 61.56%), TTS (PVE, 53.62%), AM (PVE, 46.72%), and AL (PVE, 36.45%). The polysaccharide metabolism-associated genes (*Mos_16HG008870*, *Mos_16HG008910,* and *Mos_16HG008920*) were found in *SS16.1* and may have an effect on SS content. Additionally, two co-localized QTL regions *SU3.1/SS3.1* and *SU11.1/SS11.1* were identified. This co-localization suggests a shared genetic basis for SU and SS content. The *FRT19.1* overlapped with 14 annotated genes, among which *Mos_19HG002230* and *Mos_19HG002280* may contribute to FRT synthesis and impact FRT content. To identify the candidate gene, we examined the expression patterns of two genes using previous RNA-Seq data from parental lines. *Mos_19HG002230* had little expression in mature fruits (Fig. S19A). In contrast, *Mos_19HG002280*, which encodes an E3 ubiquitin-protein ligase, could be the candidate. Expression analysis revealed that *Mos_19HG002280* transcript levels were low in young fruits but increased significantly during fruit maturation (Fig. S19B). Notably, in mature fruits, the expression of *Mos_19HG002280* was significantly higher in the paternal line Q2305 (low FRT content) compared to the maternal line Q2304 (high FRT content). The results suggest that *Mos_19HG002280* may act as a negative regulator of FRT accumulation in pumpkin fruit. It was reported that the E3 ubiquitin ligase COP1 inhibits sugar and anthocyanin accumulation in strawberry fruit [33].

TP, PR, SP, and CF were categorized as pectin and fiber traits, with 6, 10, 2, and 9 QTLs being detected, explaining 26.37%, 59.20%, 5.38%, and 66.29% of the PVE, respectively. The *SP15.1* was overlapped with *SC15.1*, implying conserved gene functions across pectin and pigment-associated pathways. A total of 13 QTLs were identified for pigment trait LU and showed significant additive and dominant effects. Three QTLs were detected for CA, with a total PVE of 14.08%. Notably, the *SC11.1* is fully within *LU11.1*, and both regions include a P450 family gene (*Mos_11HG016770*).

## Discussion

The high-quality genome provides fundamental basis for efficient and accurate genetic breeding research [34]. Thus far, only one complete genome has been published within the *Cucurbita* genus [19], which belongs to a different species from the current assembly. Other sequenced *Cucurbita* genomes rely on next-generation sequencing (NGS) technology, leaving inevitable numerous gaps. In this study, we integrated HiFi, ultra-long ONT, and Hi-C sequencing to *de novo* assemble a T2T genome for *C. moschata*. The genome has 40 telomeres across 20 chromosomes, with 10 chromosomes consisting of single contigs. The contig N50 increased from 43 Kb to 11.2 Mb, indicating a significant improvement in genome continuity and completeness compared to the reference genome Rifu [20].

With the development of sequencing technology, the long-read sequencing has been applied in numerous studies and powers the assembly of more complete genomes, like *Arabidopsis*, rice and watermelon [21, 35, 36]. The YGX2019 genome displayed superior performance in gap filling and repeat sequence resolution compared with the previously sequenced *C. moschata* genome (Rifu). It was found that 91.96% additional sequences are repetitive elements, and 52.14% are transposons. The result highlights the advantages of long-read sequencing technology in identifying repetitive sequences, particularly the transposons. Despite the widespread adoption of long-read sequencing for plant genome assembly, achieving complete and gap-free assemblies remains challenging [37, 38]. Either genome size or the repetitiveness of genome sequences could make the genome assembly complicating [39]. The ancient allopolyploidization event in *C. moschata* resulted in numerous highly homologous sequences despite attempts with multiple advanced assembly tools. Therefore, achieving the complete, gap-free pumpkin genome assemblies remains challenging. In the future, generating longer sequencing reads could resolve these complex repetitive regions, helping to uncover previously un-assembled genomic portions. For instance, the complete assembly of the cucumber genome required approximately 95× ONT ultra-long reads (N50 > 200 kb) combined with 70× HiFi data, illustrating the substantial resources needed for such complex genomes [40].

WGD serves as a primary driver for evolutionary innovation by providing abundant genetic redundancy for the emergence of novel traits. In our study, the *Cucurbita*-specific allotetraploidization event coincided with a massive expansion of gene families related to pectinesterase activity, cell wall remodeling, and pollen tube growth. Other horticultural crops studies demonstrate that chromosome doubling and WGD events directly induce the expansion and upregulated expression of pectin methylesterase (PME) and pectate lyase (PL) gene families, thereby regulating cell wall stiffness, environmental adaptation and fleshy fruit softening [41, 42]. Therefore, the genetic redundancy generated by this ancestral WGD likely provided the essential genetic basis for the morphological development of the characteristic large, fleshy pepo fruits in *Cucurbita*. Furthermore, the expansion of pollination and pollen tube-related genes post-WGD suggests a genomic mechanism for the rapid establishment of reproductive isolation, ensuring the independent speciation of the *Cucurbita* genus.

It is found that unaligned sequences accounted for up to 20% to 30% of the *Cucurbita* genomes, primarily consisting of repetitive sequences. The rapid amplification and bursts of TEs are major mechanisms that generate widespread SVs and genomic diversity [43]. Rather than being non-functional sequences, these widespread SVs frequently intersect with functional genes and regulatory regions, modifying gene expression to drive crop improvement traits, such as environmental adaptation and fruit quality [44]. This supports our observation that the SV-driven unaligned regions in *C. moschata* harbor genes significantly enriched in secondary metabolism and hypoxia responses. Because such extensive structural complexity and gene copy number variations are obscured when aligning against a single reference, constructing a *Cucurbita* pan-genome is necessary to capture the full genetic variations in the future.

Genome-wide duplication analysis identified various types of gene duplication in the YGX2019, including SDs, TDs, PDs, and DDs. Genes originating from TD and PD events were significantly enriched in pathways related to disease resistance, for instance, the phenylpropanoid biosynthesis pathway. Phenylpropanoid biosynthesis is critical to provide precursors for the synthesis of lignin and flavonoid, which are two vital compound classes for plant defense. Lignin forms a structural barrier against microbial infections and pest herbivory, serving as a major contributor to biotic stress resistance [45, 46]. Simultaneously, flavonoids function as antioxidants that reduce oxidative damage from reactive oxygen species (ROS) accumulation, induced by salt, drought and extreme temperature stresses [47, 48]. It was reported that TD and PD usually experience stronger selective pressures for self-defense functions compared to other duplication types [49]. Therefore, the enriched TD and PD genes in phenylpropanoid biosynthesis pathway indicates a potential evolutionary strategy for *C. moschata*. A resistance gene cluster on Chr19, which is composed of TDs, may appear to expand through the plant-pathogen ‘arms race’, enhancing the defense via gene dosage effect [50, 51].

Little research has been reported on detecting QTLs controlling fruit traits of *C. moschata.* Taking the YGX2019 genome as the reference, the study constructed a high-density genetic map comprising 7213 markers with a total length of 2538.2 cM, which significantly surpasses the previous map (2252.1 cM in coverage) [7]. Following systematic QTL scanning for 20 fruit traits, 165 QTLs (over eight QTLs per trait) were identified, which achieved a remarkable increase compared with the previous reports [7, 26]. The evaluated fruit traits mainly encompassed fruit morphology, sugar content, and nutritional quality, providing valuable resources for high-quality pumpkin breeding.

Interestingly, co-localization of QTLs controlling different traits was found in the study, a hint of the underlying complex trait regulation. For instance, the location of *SC11.1* locus was found fully within the *LU11.1* locus, and both contain a P450 family gene (*Mos_11HG016770*). It could be a representative case of pleiotropy, which a single gene has a multifunctional role. Cytochrome P450 enzymes are key drivers of CA diversity. They are structurally required for LU biosynthesis from α-carotene [52] and have been shown to modulate fruit colouration in citrus by altering CA accumulation [53]. Another overlap was observed between *SP15.1* and *SC15.1*. The co-localization could be explained by tight linkage, where two or more distinct genes, each controlling one trait, are located in close physical proximity. Further fine-mapping of the region would be required.

In addition, 165 QTLs identified in the study were systematically compared with previous reports. We found that 14 of these loci are consistent with those previously identified, including *TD12.1*, *TD15.1*, *TD19.1*, *SS3.1*, *SS4.1*, *SS8.1* and *SS9.1* [7], and *FT9.1*, *LD8.1*, *SU19.1*, *CA15.1*, *LU8.1*, *LU11.1* and *LU20.1* [26]. The overlap with previous work confirms the accuracy of QTLs based on the high-density map. The newly identified loci (151 out of 165) provide important insights into the genetic mechanisms of pumpkin fruit traits. The high-quality map will facilitate precise gene mapping for additional traits in future studies, and lay a robust foundation for unravelling the genetic basis of economically important traits in *C. moschata*.

Notably, the study identified six QTLs with PVE greater than 20% and 16 QTLs with PVE between 10% and 20%. The β-tubulin gene *Mos_14HG000790* within *TDLD14.1* (PVE 31.44%) for fruit morphology, and the AUX/IAA gene *Mos_12HG001420* within *LFT12.1* (PVE  20.60%) for longitudinal flesh thickness were identified as the candidate genes, respectively. Both genes had polymorphisms found on the promoters. The *Mos_14HG000790* potentially regulates cell elongation and fruit shape via microtubule reorientation, and *Mos_12HG001420* may suppress cell expansion through a mechanism similar to that of IAA17 in tomato [32]. Given that auxin can induce microtubule reorientation [54], we hypothesize that *Mos_14HG000790* and *Mos_12HG001420* may synergistically regulate pumpkin fruit size. Future studies employing promoter-GUS reporter assays are required to experimentally elucidate the precise molecular mechanisms by which these SVs regulate gene expression and drive fruit morphological diversity.

In conclusion, this study delivers a high-quality reference genome for *C. moschata* (YGX2019) and a corresponding high-density genetic map. Together, these resources provide a robust foundation for future research on the genetic mechanisms of important agronomic traits. The candidate genes we have identified, particularly for fruit shape and FT, are promising targets for crop improvement. However, we should acknowledge that the current supporting expression data is preliminary. Therefore, subsequent research should prioritize the validation of these findings. Ultimately, the function of the candidate gene can be confirmed through functional validation using transgenic or gene-editing approaches. Furthermore, considering that QTL effects can be influenced by genotype-by-environment interactions [55], future work leveraging multi-environment trials and genome-wide association studies (GWAS) would be invaluable for confirming the stability of these QTLs and their direct applicability in breeding programs.

## Materials and methods

### Plant materials

The pumpkin high-generation inbred line YGX2019 of *C. moschata* L., a crucial germplasm resource for high-quality and taro-like aroma pumpkin fruit breeding, was selected for genome sequencing. Fresh young leaves from the two-month-old YGX2019 plant were collected for DNA isolation. The well-developed roots, stems, leaves, female flowers, male flowers, and young fruits from the YGX2019 plant were harvested for RNA extraction. To construct the genetic map, intraspecific F_2_ populations were generated from the cross of two *C. moschata* inbred lines, Q2304 and Q2305, which differ in fruit shape and quality. Q2304 has an oblate/lantern shape. In comparison, Q2305 has a long/olive shape. In addition, they vary in multiple fruit quality traits, including TS, TTS, and TP. Q2304 and Q2305 were separately utilized as the female and male parents to generate the hybrid F_1_. F_2_ populations were produced through the self-pollination of F_1_. Leaves from one-month-old Q2304, Q2305, and 200 F_2_ individuals were collected for DNA extraction and sequencing. All materials were planted in the field at Baiyun Experimental Base, Guangzhou, China.

### DNA and RNA extraction

The genomic DNA and RNA of YGX2019 samples were separately extracted using the Genomic-tip kit and RNAprep Pure polysaccharide polyphenol plant total RNA extraction kit (TIANGEN, Germany). The concentration and purity of DNA and RNA were first measured using NanoDrop 2000 (Thermo Fisher Scientific, Waltham, MA, USA), and the quality and integrity were assessed using gel electrophoresis. The DNA concentration for genome sequencing was further analyzed by Qubit® DNA Assay Kit in Qubit® 2.0 Fluorometer (Invitrogen, USA).

### Illumina sequencing

High-quality genomic DNA of YGX2019 was randomly sheared by Covaris ultrasonic disruptor. The library was constructed using the protocol of Illumina (Illumina, San Diego, CA, USA), followed by pair-end sequencing using the Illumina Hiseq. Raw reads were cleaned to discard the low-quality reads (reads with adaptors or unknown nucleotides with more than 10%, or reads with more than 20% low-quality bases). After data filtering, clean data were used for subsequent analysis. K-mer analysis was applied to estimate the approximate characteristics of the genome.

### Pacbio HiFi sequencing

The DNA sample was used as input for library preparation. The library was generated using SMRTbell Express Template Prep Kit 2.0 (Pacific Biosciences) following the standard recommendations of the manufacturer. Briefly, gDNA was sheared into fragments to construct SMRTbell library. Sheared gDNA was concentrated using AMPure PB beads. The DNA library was loaded in SMRT Cells with the Sequencing Reagent Kit in accordance with the manual.

### ONT ultra-long sequencing

For Oxford Nanopore sequencing, the prepared library by the SQK-LSK110 ligation kit was loaded onto primed R9.4.1 Spot-On Flow Cells and sequenced using a PromethION sequencer (Oxford Nanopore Technologies, Oxford, UK) with 48 to 72 hours runs (Wuhan Benagen Technology Co., Ltd., Wuhan, China). Raw data were analyzed using the Oxford Nanopore GUPPY software (v0.3.0).

### Hi-C sequencing

The Hi-C library was constructed according to the standard protocol [56]. Tissues were ground and fixed in 4% formaldehyde solution. The cross-linking reaction was quenched by 2.5 M glycine. Samples were carefully washed and digested with restriction enzyme *Mbo I*. Mark DNA ends with biotin-14-dCTP and blunt-end ligation of the cross-linked fragments. The cross-link was reversed by proteinase K. Ends of sheared fragments were repaired by T4 DNA polymerase, T4 polynucleotide kinase and Klenow DNA polymerase. Biotin-labelled Hi-C samples were specifically enriched using streptavidin C1 magnetic beads. After adding A-tails and following ligation by sequencing adapters, the Hi-C library was amplified and sequenced on the Illumina HiSeq-2500 platform (PE 150 bp).

### RNA sequencing

The qualified mRNA were extracted from different tissues of YGX2019 (roots, stems, leaves, female flowers, male flowers and young fruits) were fragmented and used as the templates for the cDNA preparation. The equal amounts of six different cDNA samples were mixed to construct a pool. The library was constructed and sequenced on Illumina.

### Genome assembly

To estimate the size and heterozygosity of the genome, the k-mer (*k* = 21) frequency distribution was analyzed using Jellyfish (v2.3.0) (https://github.com/jamesturk/jellyfsh). For genome assembly, we used Hifiasm (0.19.8-r603) [57] to integrate HiFi reads and ultra-long ONT reads to assemble the YGX2019 genome with default parameters. We employed Hi-C data and utilized the juicer (v1.6) [58] and 3D-DNA pipeline [59] for scaffolding, and then the erroneous orders of contigs were manually adjusted in juicebox (1.11.08). The Hi-C interaction matrices were computed using HiC-Pro (v3.1.0) [60], and the Hi-C interaction heatmap was visualized using HiCPlotter with a bin size of 100 kb [61].

We aligned contigs assembled by NextDenovo (v2.5.2) [62] and HiCanu (v2.2) [63] to the genome using Minimap2 (v2.26) [64], and supplemented missing telomeres based on sequence similarity. NextDenovo was used with the parameters: input_type = raw, read_type = ont, genome_size = 300 000 000. After that, we polished the results using NextPolish (v1.4.1) [65]. We assessed the quality of the YGX2019 genome using gfastats (v1.3.6) [66] to calculate the basic information, including N50 and N90. BUSCO (v5.5.0) with the library of embryophyta_odb10 was used to assess the completeness of the genome. QV value was calculated using Merqury [67]. We mapped HiFi and ONT reads to the genome to check the read coverage.

### Genome annotation

Repetitive sequences were first predicted using RepeatModeler (v2.0.5) [68] and then masked using RepeatMasker (v4.1.2-p1) (https://www.repeatmasker.org/) to annotate TEs. The tRNA sequences were predicted using tRNAscan-SE (v2.0.12) [69], while other non-coding RNAs, including miRNA, lncRNA, and rRNA, were identified with the Rfam database (v14.10) using Infernal (v1.1.5). Gene prediction was performed on the masked genome using the BRAKER3 pipeline (v3.0.8) [70–72], which integrates several bioinformatics tools. For the *ab initio* prediction method, AUGUSTUS was trained within the pipeline, and BLAST was used to filter out redundant training gene structures [73, 74]. To incorporate evidence from both protein data and short-read RNA-Seq data, SAMtools [75, 76] were employed to process RNA-Seq alignment results in BAM file format. StringTie2 [77] was used for transcriptome assembly, and GffRead [78] was employed to check and correct transcript files. Using the complementary information from RNA-Seq data and protein alignment results, BEDTools [79] analyzed overlapping regions to optimize the gene structure models. GeneMark-ETP [80] further integrated RNA-Seq and protein data. Finally, TSEBRA [81] was used to combine all available evidence. The homologous protein library utilized was Eukaryota.fa, provided by BRAKER3. RNA-Seq data were aligned to the genome using HISAT2 (v2.2.1) [82] before being processed with BRAKER3. Genes lacking start or stop codons were removed using GffRead, and the annotation was refined with AGAT (agat_convert_sp_gxf2gxf.pl) (https://github.com/NBISweden/AGAT) to remove redundant features and ensure a complete, well-ordered annotation result.

Functional annotation of protein-coding genes was performed using BLAST+ (v2.15) and diamond (v2.1.9) [83] with the parameter ‘e value^1e-5^’ to compare the protein sequences against the databases. The NCBI non-redundant protein database (NR) was taken for gene alignment using diamond. The Uniprot-sprot and Uniprot-trembl database was employed for gene alignment using BLAST. InterProScan (v5.67–99.0) was applied to identify protein domains by comparing the protein sequences against multiple protein domain databases, including Pfam, SMART, PANTHER, AntiFam and FunFam database. GO terms and KEGG pathways were obtained using eggNOG-mapper [84].

### Genome quality assessment

SyRI (v1.6.3) [85] was used to identify regions of synteny, inversion, translocation, and duplication. The genome-specific sequence types which refer to non-syntenic regions and do not include inversion, translocation, or duplication, were annotated using the same method as genome annotation. Proteome (protein-coding gene repertoire) quality assessment alignment was performed using OMArk (v0.3.0) [86]. Liftoff (1.6.3) [87] was employed to transfer the gene structure annotation results from the YGX2019 genome to others to assess the migration status.

### Comparative genomic analysis

To identify genomic synteny and SVs, whole-genome alignments between YGX2019 and other published genomes were performed using Minimap2 (v2.30-r1287) [64]. Synteny blocks, SVs and unaligned regions were identified using SyRI (v1.6.3) [85]. The synonymous substitution rates (*K_s_*) of these collinear gene pairs were calculated using WGDI (v0.75) [88]. Protein sequences from nine plant species were clustered into orthologous gene families using OrthoFinder (v2.5.5) [89]. Single-copy orthologous genes were aligned using MAFFT (v7.525) [90] and used to construct a maximum-likelihood phylogenetic tree via IQ-TREE (v3.0.1) [91]. Divergence times among species were estimated using MCMCTree (v4.10.10) [92]. Based on the time-calibrated tree and orthogroup counts, the expansion and contraction of gene families across lineages were inferred using CAFE5 [93]. Gene ontology (GO) enrichment analyses for the significantly expanded gene families and the genes located within the unaligned sequences were performed using the R package clusterProfiler. GO terms with an adjusted *P* value <0.05 were considered significantly enriched.

### Identification of SDs and duplicated genes

SDs were identified using biser (v1.4) [94] on the genome that had been soft-masked for repetitive sequences with RepeatMasker, filtering out sequences shorter than 1 Kb. The total SD length for the genome was calculated after removing overlapping regions. The duplicated genes were identified and classified using the doubletrouble R package [95]. Paralogs that are anchor pairs in syntenic regions are classified as SDs. Among the remaining duplicates, gene pairs that are physically adjacent to each other in the genome are classified as TDs, those separated by up to 10 intervening genes are classified as PDs, and all other duplicates are classified as DDs. The variation of duplicated genes was identified by JCVI [96]. The KEGG enrichment of duplicated genes was employed by R enricher function.

### Phenotypic variation in the F_2_ population

The assessment of 20 fruit related traits of Q2304, Q2305, and generated F_2_ populations was applied. Briefly, mature fruits were harvested from the field. Fruit skin colour was investigated. FW, FT, LFT, TD, and LD were measured. The TDLD ratio was calculated. SS were estimated using the saccharimeter. Other 12 traits were monitored using kits according to the corresponding protocols of manufacturers, including TS (Solarbio®, Total Carbohydrate Content Assay Kit, BC2710), FRT (Solarbio®, Plant Tissue Fructose Content Assay Kit, BC2450), SU (Solarbio®, Plant Sucrose Content Assay Kit, BC2460), TTS (Solarbio®, Total Starch Content Assay Kit, BC0700), AM (Solarbio®, Amylose Starch Content Assay Kit, BC4260), AL (Solarbio®, Amylopectin Starch Content Assay Kit, BC4270), TP (Solarbio®, Pectin Content Assay Kit, BC1400), PR (Solarbio®, Protopectin Content Assay Kit, BC3680), SP (Solarbio®, Soluble Pectin Content Assay Kit, BC4120), and CA (Solarbio®, Plant Carotenoid Content Assay Kit, BC4330), CF (mlbio, Plant Crude fibre ELISA Assay Kit, YJ276025), and LU (mlbio, Plant Lutein ELISA Assay Kit, YJ270634). The distributions of all pumpkin fruit traits were displayed using the histogram and density plots. R was applied to calculate the Pearson correlation coefficients of different traits.

### DNA isolation and sequencing

The phenol-chloroform-isoamyl alcohol extraction method was applied to obtain pure DNA of Q2304, Q2305 and 200 F_2_ individuals. Generally, the extraction buffer (phenol-chloroform-isoamyl alcohol) was added to the ground leaves. After ethanol precipitation and gentle wash, the DNA pellet was resuspended in the EB buffer. The DNA concentration and purity of all samples were measured using NanoDrop (Thermo Fisher Scientific, Waltham, MA, USA), and the quality and integrity were assessed using the 1% agarose gel electrophoresis experiment. DNA samples were stored at minus 20°C for subsequent experiments.

Library preparation is performed using the BGI Optimal DNA Library Prep Kit (BGI-Shenzhen, China). Next, the final double-strand library products are denatured to generate the single-stranded library product. Then, the circularization reaction is set up to get single-stranded circularized DNA products. Any single-stranded linear DNA will be digested to remove. The final single-stranded circularized library is amplified with phi29 and rolling circle amplification (RCA) to generate the DNA nano ball (DNB), which carries about 300 copies of the initial single-stranded library molecule. The DNBs are loaded into the patterned nanoarray and sequencing reads of PE150 reads length are generated with the DNBSEQ-G400 platform (BGI-Shenzhen, China).

### SNP discovery and genotyping

The raw data was subjected to quality control using fastp (0.23.2) [97] to remove low-quality reads. The reference genome used for sequence alignment was the genome assembled in the study (YGX2019.fa). The filtered clean reads were aligned to the reference genome using the BWA-mem2 algorithm (2.2.1). Variant detection for the population was performed chromosome-by-chromosome using Bcftools (1.6) [98] mpileup. The VCF files for all chromosomes were then merged using Bcftools concat, producing the final variant dataset. SNP filtering was carried out using GATK4 (4.2.3.0) [99] SelectVariants module, with parental genotypes serving as the filtering criteria.

### Genetic linkage map construction

The genetic map was constructed using Lep-Map3 software. Initially, the ParentCall2 module was used to filter non-informative markers. Subsequently, the SeparateChromosomes2 module was applied to assign the remaining high-quality SNP locations into the distinct linkage groups. SNPs within each linkage group were then ordered, and OrderMarkers2 module was employed to calculate the Kosambi genetic distances between adjacent markers. Finally, the distinct function from the R/dplyr package was applied to group non-recombinant SNPs into bins. The ID of the first SNP was used to represent the bin.

### QTL mapping

QTL analysis was conducted using the CIM method implemented in the R/QTL package [100]. A permutation test with 1000 iterations was performed to establish the significance threshold for QTLs (*P* < 0.05). The confidence interval of QTL was defined using a 1.5-LOD support interval. The fitqtl function in R/qtl was employed to estimate the additive effects of QTLs and calculate the PVE by each locus. Fruit traits QTL naming followed the nomenclature recommendations in cucumber [101]. The cis-regulatory elements were identified using PlantCARE [102].

### Indel molecular marker development

The major-effect QTL of the targeted trait was selected. Variants were searched in the QTL mapping interval based on the genome information and resequencing data of two parental lines, and chosen as the sites for Indel molecular marker development. Primer Premier software was used to design the Indel primers according to these variants. After PCR amplification, the products from the two parents and F_1_ were firstly examined through electrophoresis and silver staining. The bands were recorded and analyzed. Markers with clear bands and good polymorphism were screened and then validated in the F_2_ population. Individuals with bands that were separately identical to those of Q2304 and Q2305 were classified as A and B types. Those with both A and B band types were classified as H type. Genotyping was conducted in the F_2_ population, and combined with the phenotypic data of the targeted trait for further analysis. The concordance rate between the genotype and phenotype was analyzed to evaluate the linkage of the Indel marker and the trait. A high concordance rate indicates the accuracy of the QTL, and the Indel marker could be utilized for the genotype-based prediction of the phenotype.

### KASP marker development

Based on the interval of *LFT12.1*, KASP markers were developed outside and within the candidate region according to the polymorphic SNPs between two parents (Table S20). F_2_ individuals were selected for genotype–phenotype analysis to narrow the mapping interval. The KASP assay was performed according to the competitive allele-specific PCR primer amplification refractory mutation system [DNA template (10–100 ng), allele 1 primer (0.15 μl), allele 2 primer (0.15 μl), common primer (0.4 μl), 2× PARMS master mix (5 μl), and H_2_O]. The cycling conditions were set as previously described [103].

### Quantitative real-time PCR analysis

To validate the expression patterns of candidate genes, RNA was extracted from various tissues and fruits at different developmental stages using the TRIzol reagent. cDNA was synthesized using the TransScript® One-Step gDNA Removal and cDNA Synthesis SuperMix (TransGen Biotech, Beijing, China). Gene-specific primers were designed using Primer Premier 5 (Table S21). The actin gene (*Mos_11HG005290*) served as the internal control for normalization. Quantitative real-time PCR (qRT-PCR) was performed on a CFX96 Real-Time PCR Detection System (Bio-Rad, CA, USA) following cycling conditions described previously [104]. Relative gene expression levels were calculated using the 2^−ΔΔCt^ method.

## Supplementary Material

Web_Material_uhag141

## Data Availability

The raw reads generated in this study have been deposited in the NCBI sequence read archive (SRA) with the accession number Sequencing Data PRJNA1299617. The genome file has been deposited in the CNCB Genome Warehouse under accession number GWHGPXY00000000.1.
