# Supplementary material for: Telomere-to-telomere genome assembly of pumpkin (Cucurbita moschata) facilitates QTL identification of fruit traits
Source: Hortic Res. 2026 Apr 16;13(8):uhag141. doi: 10.1093/hr/uhag141 (PMC13454485; doi:10.1093/hr/uhag141)
Supplement: Supplementary file 1 [file web_material_uhag141.zip › Supplementary Figures.docx]

**Supplementary Figures**

**Figure S1. Genome survey of YGX2019 based on 21-mer length analysis.**

**Figure S2. The length distribution of contigs assembled by Hifiasm, Nexdenovo, and Canu.**

**Figure S3. Hi-C heatmap of 20 scaffolds**

**Figure S4. Flowchart showing the method used for assembling the YGX2019 genome.**

**Figure S5. The collinearity between contigs and scaffolds enables the contigs to fill all missing telomeres**

**Figure S6.**

**A** Genomic synteny and structural variations between *C. moschata* (YGX2019) and *C. pepo* (MU-CU-16)

**B** Genomic synteny and structural variations between C. moschata (YGX2019) and *C. arg* (SMH-JMG-627)

**C** Genomic synteny and structural variations between C. moschata (YGX2019) and *C. arg sororia* (sororia)

**Figure S7.**

**A** Gene Ontology (GO) enrichment analysis of the genes in unaligned sequence between YGX2019 and HZAU.

**B** Gene Ontology (GO) enrichment analysis of the genes in unaligned sequence between YGX2019 and MU-CU-16.

**C** Gene Ontology (GO) enrichment analysis of the genes in unaligned sequence between YGX2019 and SMH-JMG-627.

**D** Gene Ontology (GO) enrichment analysis of the genes in unaligned sequence between YGX2019 and sororia.

**Figure S8. Gene Ontology (GO) enrichment analysis of the significantly expanded gene families in *C. moschata* (YGX2019).**

**Figure S9. The number of different duplication mode genes in YGX2019 and Rifu genomes.**

**Figure S10. The top five significant KEGG pathways enriched in duplicated genes.**

**A** The top five significant KEGG pathways enriched in DD genes.

**B** The top five significant KEGG pathways enriched in PD genes. The p values were corrected using the FDR method.

**Figure S11. Histogram and density plots of pumpkin fruit traits and their correlation matrix**

**Figure S12. The synteny between linkage map and physical map.**

**Figure S13. The Indel molecular marker that is closely linked to the TDLD major QTL, and the application of the marker in the assessment of the fruit trait of the F_2_ population**

**Figure S14. Gene expression profiles in major-effect QTL interval during early fruit development (-2 d to 5 d) in Q2304. -2 d represents 2 days before flowering, 0 d represents day of flowering, and 5 day represents 5 days after flowering.**

**A** Expression of genes in *TDLD14.1* interval.

**B** Expression of genes in *LFT12.1* interval.

**Figure S15. Expression analysis of key candidate genes via qRT-PCR.**

**A** Relative expression of the TDLD candidate gene *Mos_14HG000790* in different pumpkin varieties. Varieties Q2302–Q2304 exhibit oblate/lantern shapes, while Q2305–Q2307 exhibit long/olive shapes. The orange and green colors separately indicate the relative expression of the corresponding candidate gene and the value of phenotype.

**B** Tissue-specific and fruit developmental expression pattern of *Mos_14HG000790* in Q2304.

**C** Relative expression of the LFT candidate gene *Mos_12HG001420* across pumpkin varieties with contrasting fruit shapes. The orange and green colors separately indicate the relative expression of the corresponding candidate gene and the value of phenotype.

**D** Tissue-specific and fruit developmental expression pattern of *Mos_12HG001420* in Q2304.

**Figure S16. The variations within the upstream 2 kb region of candidate genes transcription start site between the parent lines.**

**A** The variations within the upstream 2 kb region of the *Mos_14HG000790* transcription start site between the parent lines.

**B** The variations within the upstream 2 kb region of the *Mos_12HG001420* transcription start site between the parent lines.

**Figure S17. Fine mapping of the candidate gene for LFT. The left sides are two parents, F_1_ and F_2_ recombinants. The right sides are the corresponding phenotypes (LFT) of these left side plants.**

**Figure S18. KASP genotyping scatter plots.**

**A** Genotype clustering results for marker M794105. Samples clustered along the X-axis with blue fluorescence represent the FAM homozygous genotype (thick-flesh phenotype), whereas samples clustered along the Y-axis with green fluorescence represent the HEX homozygous genotype (thin-flesh phenotype). Samples distributed between the two clusters with red fluorescence indicate heterozygous genotypes (intermediate phenotype). Gray dots represent controls.

**B** Genotype clustering results for marker M876100. Samples clustered along the X-axis with blue fluorescence represent the FAM homozygous genotype (thin-flesh phenotype), whereas samples clustered along the Y-axis with green fluorescence represent the HEX homozygous genotype (thick-flesh phenotype). Samples located between the two clusters with red fluorescence indicate heterozygous genotypes (intermediate phenotype). Gray dots represent controls.

**Figure S19. Expression analysis of two candidate genes controlling *FRT19.1* in fruits.**

**A** Expression of *Mos_19HG002230* in young (YF) and mature fruits (MF) of Q2304 and Q2305, respectively.

**B** Expression of *Mos_19HG002280* in young (YF) and mature fruits (MF) of Q2304 and Q2305, respectively.

* indicates p < 0.05, ** indicates p < 0.01
